# A machine learning-based online prediction model for recurrence risk in patients with *Klebsiella pneumoniae* liver abscess: a multicenter retrospective study

**DOI:** 10.3389/fcimb.2026.1830022

**Published:** 2026-05-20

**Authors:** Liyong Zhang, Ying Wang, Yanchao Dong, Yihao Qu, Yuwei Fu, Jiaqi Chen, Kai Chen, Jinhua Cui, Ziyu Bai, Jian Li, Aijun Yu

**Affiliations:** 1 The First Department of General Surgery, Affiliated Hospital of Chengde Medical University, Chengde, Hebei, China; 2 Department of Hepatobiliary Surgery, Kailuan General Hospital, Tangshan, Hebei, China; 3 Department of Interventional Therapy, First Hospital of Qinhuangdao, Qinhuangdao, China

**Keywords:** *Klebsiella pneumoniae*, liver abscess, machine learning, prediction model, recurrence, SHAP

## Abstract

**Objective:**

*Klebsiella pneumoniae* liver abscess (KPLA) has a non-negligible risk of recurrence after treatment, imposing a substantial burden on affected patients and the global healthcare systems. To address this issue, we developed and validated a machine learning model that integrates patients’ clinical data and laboratory indicators for early risk prediction of KPLA.

**Methods:**

This multicenter retrospective study included 829 KPLA patients from three tertiary hospitals (2016–2024). Data of 722 patients from the Affiliated Hospital of Chengde Medical University and Kailuan General Hospital were divided into a training set (n = 506) and an internal testing set (n = 216), and the data of the 107 patients from the First Hospital of Qinhuangdao were included in the quasi-external validation set. Twenty-four candidate variables were collected, and 9 key predictors were retained based on the results of univariate analysis, Least Absolute Shrinkage and Selection Operator regression, and the Boruta algorithm. After constructing seven machine learning models, with the logistic regression model (LM) as the baseline control, through accuracy and area under the curve (AUC), decision curve analysis, and calibration curve analysis, the extreme gradient boost method (XGBoost) was selected as the final prediction model. SHAP (SHapley Additive exPlanations) analyses were conducted to enhance model interpretability, and a web-based tool was developed for use in clinical practice.

**Results:**

The hyperparameter-optimized XGBoost model showed optimal performance, with AUC values of 0.936 (95% CI: 0.914–0.959), 0.868 (95% CI: 0.799–0.938), and 0.904 (95% CI: 0.819–0.988) in the training, internal testing, and quasi-external validation sets, respectively. The intersection results of the three abovementioned feature selection approaches yielded 9 key predictors, including age, type 2 diabetes mellitus, malignant neoplasm, biliary disease, fibrinogen level, procalcitonin level, multiple abscesses, septic shock, and Sequential Organ Failure Assessment (SOFA)-2 score. The web-based tool enabled to assess individualized recurrence risk.

**Conclusions:**

The XGBoost-based prediction model integrates clinical and laboratory indicators to accurately predict KPLA recurrence risk, with good generalizability and interpretability. The accompanying web-based tool provides a practical decision-making application for clinicians to identify high-risk patients early and implement personalized interventions.

## 1 Introduction

*Klebsiella pneumoniae* liver abscess (KPLA) is the predominant form of pyogenic liver abscess (PLA). Hypervirulent *K. pneumoniae* strains frequently cause invasive KPLA syndrome (IKPLAS) accompanied by distant organ metastases, which significantly increases patient disability and mortality (Gu et al., 2025; Zhang et al., 2025a). The standard treatment for KPLA is antimicrobial therapy combined with drainage, which achieves clinical cure in most patients. Nonetheless, KPLA carries a non-negligible recurrence risk of 18%–25% after treatment (Wang et al., 2022; Zhang et al., 2025b). Recurrent KPLA results in prolonged hospitalization, increased medical costs, progressive liver tissue damage, and potentially life-threatening complications such as sepsis, imposing a heavy burden on patients and healthcare systems.

Currently, the clinical assessment of KPLA recurrence risk mainly depends on traditional clinical indicators and physician experience, including age, comorbidities, abscess number, septic shock, and inflammatory markers (Lee et al., 2021; Wang and Xue, 2023; Zhang et al., 2025b). However, these methods have critical limitations: KPLA recurrence is a multifactorial process that cannot be fully quantified by few indicators (Rossi et al., 2022; Cui et al., 2025); traditional assessment is subjective and lacks standardized quantitative criteria. Recent studies on KPLA recurrence prediction remain insufficient. Zhang et al (Zhang et al., 2025b). developed a KPLA recurrence model but was limited by single-center design and traditional logistic regression. Cui et al (Cui et al., 2025). identified recurrence risk factors but failed to construct a predictive model. Traditional logistic regression can handle nonlinear and high-dimensional features via polynomial transformation and LASSO regularization, but performs poorly in fitting complex interactions of KPLA recurrence. Moreover, most previous studies lacked independent external validation. Thus, existing researches cannot meet the clinical need for accurate and individualized KPLA recurrence risk evaluation.

By processing high-dimensional data and capturing complex nonlinear correlations, machine learning (ML) methods can outperform traditional statistical approaches in achieving high predictive accuracy (Rajula et al., 2020; Pfob et al., 2022; Sheth et al., 2023). Explanatory methods such as SHapley Additive exPlanations (SHAP) and Local Interpretable Model-Agnostic Explanations have been introduced to resolve the issue of limited transparency (Rodríguez-Pérez and Bajorath, 2020; Bifarin, 2023). Despite their robust predictive capabilities stemming from inherent complexity, ML methods are often labeled as “black boxes” (Kropiunig and Sørensen, 2025; Moriya et al., 2025) because of the poor interpretability of their internal mechanisms, which limits their integration into clinical practice. Additionally, many ML studies are constrained by single-center data, a relatively small sample size, and a lack of external validation, which reduces the generalizability of these ML models.

To overcome these limitations, we conducted a multicenter retrospective study using electronic medical records (EMRs) of patients with PLA from three tertiary hospitals. The study aimed to develop and validate an interpretable ML model to predict KPLA recurrence, identify the key risk factors, and validate the top-performing model through SHAP analysis. This model was subsequently developed into a web-based online tool to enable early individualized risk assessment and support the clinical management of patients with KPLA.

## 2 Methods

### 2.1 Study population

This multicenter retrospective study consecutively included data of patients with KPLA admitted to the following three hospitals between January 2016 and December 2024: Affiliated Hospital of Chengde Medical University, Kailuan General Hospital, and First Hospital of Qinhuangdao. Patient data from The First Department of General Surgery of Affiliated Hospital of Chengde Medical University and the Department of Hepatobiliary Surgery of Kailuan General Hospital were used for model training and internal testing, while patient data from the Department of Interventional Therapy of First Hospital of Qinhuangdao were used for external validation.

This study complies with the Declaration of Helsinki and was approved by the institutional review boards of the participating hospitals (Approval number: CYFYLL2025552). Because of the retrospective study design and the use of deidentified patient information, the requirement for informed patient consent was waived.

### 2.2 Inclusion and exclusion criteria

**Inclusion criteria:** (1) clinical symptoms, including fever, vomiting, jaundice, and upper abdominal pain; (2) radiological confirmation of hepatic abscesses or lesions through ultrasonography, computed tomography (CT), or magnetic resonance imaging; (3) positive blood or abscess cultures or a therapeutic response to antibiotics; and (4) definitive diagnosis confirmed through percutaneous or surgical drainage. PLA diagnosis was confirmed when criteria (1) and (2) and at least one item from criteria (3) and (4) were met. The inclusion criteria for KPLA were as follows: (1) confirmed diagnosis of PLA and (2) positive *K. pneumoniae* culture from blood or abscess samples.

**Exclusion Criteria:** (1) age < 18 years; (2) incomplete clinical or imaging data.

### 2.3 Data collection and preprocessing

Demographic and clinical data were collected from the medical records of patients at the time of initial admission. From the collected data, 24 variables were finally included in the analysis: (1) general information: age and gender; (2) medical history: presence of type 2 diabetes mellitus (T2DM), hypertension, malignant neoplasm, heart disease, cerebral infarction, and biliary disease; (3) initial laboratory parameters at admission: alanine aminotransferase, fibrinogen (FIB), albumin (ALB), platelet, procalcitonin (PCT), C-reactive protein (CRP), and white blood cell (WBC) count; (4) imaging findings: location, number, and diameter of abscesses and presence of gas-containing abscesses; (5) complications: septic shock, pneumonia, or pleural effusion; and (6) treatment: antibiotic therapy alone or antibiotic therapy combined with drainage. The Sequential Organ Failure Assessment-2 (SOFA-2) score, an updated quantitative measure of organ dysfunction, was additionally calculated for each patient. The scoring criteria are shown in **Supplementary Figure 1**.

Feature selection plays a crucial role prior to model training, as it effectively reduces noise and avoids overfitting by eliminating irrelevant and redundant features. We utilized the following specific criteria for excluding variables: (1) variables with a missing rate exceeding 20% (uric acid, blood glucose, and blood urea nitrogen) and (2) variables deemed to show no significant association with recurrent liver abscess based on prior research and clinical expertise. Variables including blood glucose, blood urea nitrogen, and uric acid were excluded due to a missing rate > 20%, to avoid biased results from excessive imputation. Although these variables are closely associated with metabolic status, renal function, and inflammatory response, which are potentially relevant to KPLA recurrence, they were not included in the model owing to excessive missing data. For PCT and CRP with low missing rates (≤20% missing values), multiple imputation was performed using the MiceForest package within the R package. **Supplementary Figure 2** shows the missing status of relevant variables.

### 2.4 Outcome variables: diagnosis of recurrent KPLA

Recurrent KPLA was defined as a new episode meeting the diagnostic criteria for KPLA, occurring at least 4 weeks after the completion of treatment for the initial episode and the achievement of clinical cure (Solomkin et al., 2010; Rossi et al., 2022). Clinical cure was defined as the resolution of clinical symptoms (e.g., fever, abdominal pain), radiological evidence of abscess resolution (complete resolution or residual lesion size <1 cm on imaging), and normalization of inflammatory markers (e.g., C-reactive protein, white blood cell count). All patients were followed up for a minimum of 12 months to capture recurrence events.

### 2.5 Feature selection and model construction

During model development, the study data were randomly stratified into the training set (70%) and testing set (30%). Potential predictors in the training set were identified by screening baseline variables with three independent methods: univariate analysis, least absolute shrinkage and selection operator (LASSO) regression, and Boruta algorithm. The univariate analysis is a classical selection method based on P values. Variables with a P-value of <0.05 were considered statistically significant and extracted. The LASSO regression model identifies features with non-zero coefficients as potential predictors. It can eliminate multicollinearity and avoid overfitting of variables. We used LASSO regression combined with 10-fold cross-validation to screen variables from the baseline high-dimensional data. Boruta algorithm, a feature selection method based on the variable importance measure, identifies critical features by comparing the Z-values of candidate features with that of “shadow features.” The Z-value of each real feature is obtained using a random forest (RF) classifier in each iteration, and the Z-value of each shadow feature is created by random shuffling of the real features. The algorithm can iteratively remove features showing less relevance than random shadow features. Thus, by performing multiple internal bootstraps, the algorithm retains only those relevant features with Z-values higher than the maximal Z-value of shadow features. The intersection of three methods was adopted to avoid data leakage caused by single univariate filtering, and to retain features with both statistical significance and clinical importance.

Seven supervised ML algorithms, including logistic regression model (LM), decision tree (DT), random forest (RF), eXtreme gradient boosting (XGBoost), light gradient boosting machine (LightGBM), categorical boosting (CatBoost), and Multilayer Perceptron (MLP), were used to construct prediction models. Hyperparameter optimization was performed using stratified 5-fold cross-validation on the training set with the area under the receiver operating characteristic curve (AUC) as the optimization objective. For tree-based models (decision tree, random forest, CatBoost) and MLP, hyperparameters were tuned via grid search, while random search (50 iterations) was used for XGBoost and LightGBM to efficiently explore high-dimensional parameter spaces. Key optimized parameters included tree depth, learning rate, number of trees, minimum samples per node, and feature sampling proportion. The optimal parameter combination was selected to train the final model for improved generalization and reduced overfitting.

Model performance on training, testing, and external validation sets was thoroughly evaluated using the following metrics: accuracy, F1 score, sensitivity, specificity, precision (positive predictive value, PPV), negative predictive value (NPV), and the maximum area under the curve (AUC) of the receiver operating characteristic (ROC) curve. To further assess the clinical utility of the models, decision curve analysis (DCA) and calibration curves (CC) were employed as part of a comprehensive evaluation. DCA quantifies the net benefit across varying risk thresholds and provides an intuitive measure of the practical utility of the model in guiding clinical decision-making. The positioning of the model’s decision curve above the “treat all” and “treat none” baselines suggests that it effectively reduces the risk of overtreatment and missed diagnoses, thus minimizing associated clinical harms. Similarly, CC assesses the extent to which predicted probabilities align with actual outcomes, with the predictor line closer to the diagonal line indicating better calibration, an essential criterion for personalized risk prediction. By combining traditional performance metrics with DCA-derived clinical application and CC-based reliability, this three-pronged evaluation framework supports the selection of models that balance predictive accuracy with real-world clinical applicability.

### 2.6 Model interpretability

SHAP is utilized for explaining ML model predictions. Based on the Shapley value from game theory, SHAP is applied to ML to quantify each feature’s contribution to model prediction. The size of the SHAP value reflects the critical nature of a feature in model prediction. A larger SHAP value implies a greater impact of the feature on the prediction, indicating that it has a crucial role in the model; conversely, a smaller SHAP value shows a lesser impact of the feature on the model. SHAP values can also be positive or negative. Positive values indicate a positive impact of the feature on prediction, which increases the predicted value, while negative values indicate a negative impact, which lowers the predicted value. Here, we reported feature importance for determining the optimal model. Based on SHAP values, we performed descriptive quantitative ranking of feature importance; potential inter-correlations and confounding among predictors (e.g., T2DM and age, septic shock and SOFA-2 score) were considered during interpretation. We strictly distinguished statistical associations from clinical causal plausibility to avoid over-interpretation of model outputs.

### 2.7 Web application

To facilitate the model’s utilization in research and preliminary clinical reference, we developed a publicly accessible web-based prediction platform using Shiny (https://kplariskpredict.shinyapps.io/xgboost/), with the source code deposited in GitHub. This platform integrates SHAP interpretability analysis to display feature importance, helping users intuitively identify key factors associated with KPLA recurrence risk. Notably, this tool is an exploratory research prototype constructed based on retrospective multicenter data and has not been prospectively validated in clinical practice. It is intended for research use only and cannot replace professional clinical decision-making.

### 2.8 Statistical analysis

Data analysis was performed using R software (version 4.4.2) and SPSS (version 27.0). Continuous variables are expressed as median and interquartile range (IQR), and their distribution was assessed using the Kolmogorov–Smirnov test. Continuous variables were compared using the Mann–Whitney *U* test or the Kruskal–Wallis H test, as appropriate. Categorical variables are expressed as counts with percentages and compared using the chi-square test or Fisher’s exact test. A two-tailed P-value of <0.05 was considered statistically significant.

## 3 Results

### 3.1 Details of patient cohorts and baseline characteristics

**Figure 1:**
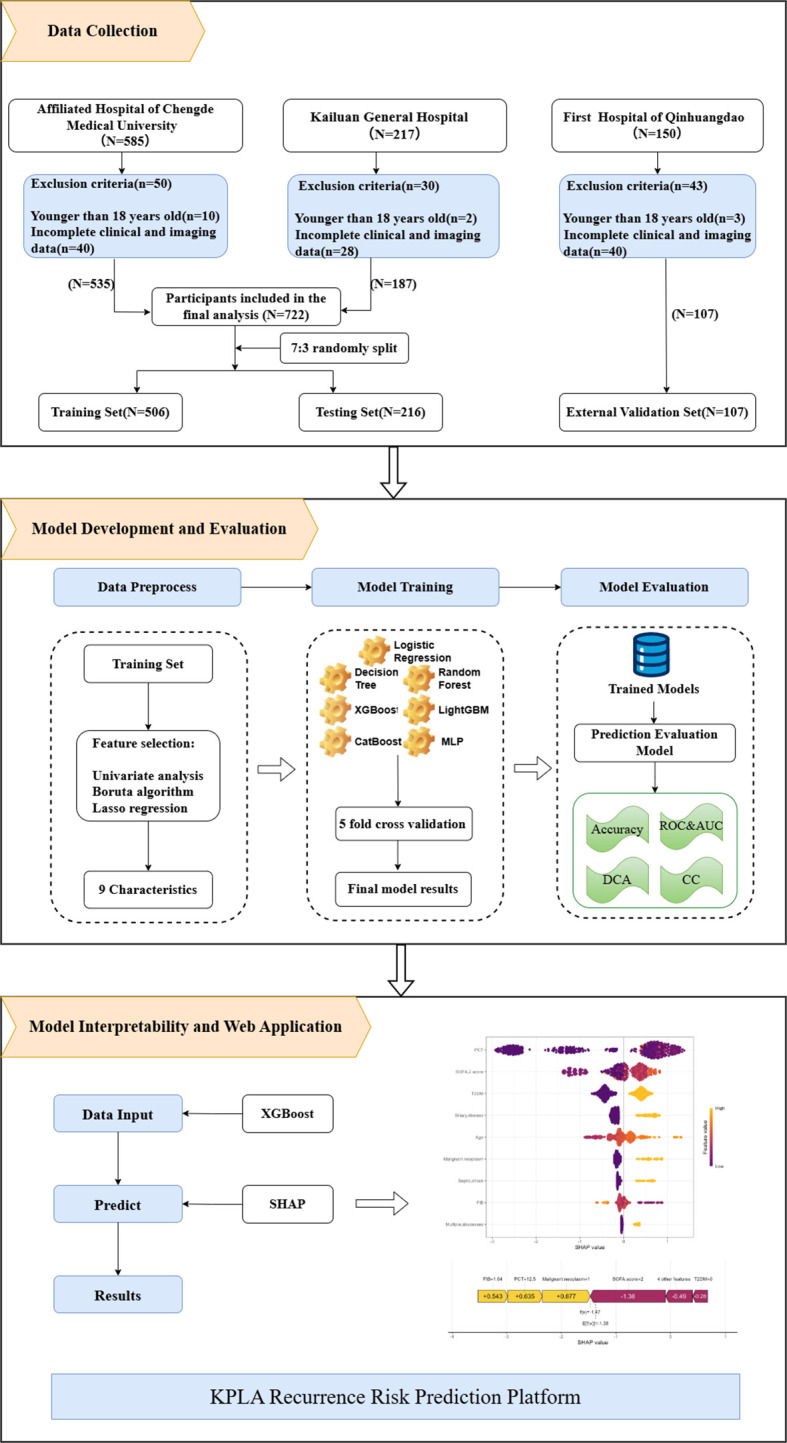
The overall study workflow. ROC, receiver operating characteristic; AUC, area under the curve; DCA, decision curve analysis; CC, calibration curve; SHAP, SHapley Additive exPlanations.

**Figure 1** shows the overall study workflow. A total of 829 patients with KPLA were recruited from three tertiary medical centers. Of these, 722 patients from two centers were randomly assigned to the training set (n = 506) and the testing set (n = 216) in a 7:3 ratio. The remaining 107 patients from the First Hospital of Qinhuangdao were included in the quasi-external validation set.

**Table 1** summarizes baseline demographic and clinical characteristics of the three cohorts. Overall, 21.95% of patients (n = 182) experienced KPLA recurrence, while the remaining 78.05% (n = 647) had no recurrence. Male patients accounted for 63.21% of the total cohort, with no significant variation in sex distribution across the cohorts. Regarding medical history, the prevalence of T2DM, malignant neoplasms, and biliary disease was significantly higher in the recurrence group than in the non-recurrence group (all P < 0.001). Regarding laboratory parameters, the recurrence and non-recurrence groups showed significant differences in the levels of FIB (P = 0.017), ALB (P = 0.013), and PCT (P < 0.001). Additionally, significant differences were observed in abscess number (P = 0.032), septic shock occurrence (P < 0.001) and SOFA-2 score (P < 0.001) between the two groups.

**Table 1:** Comparison of baseline characteristics and clinical data between the recurrence and non-recurrence groups.

Variables	Training set (n = 506)			Testing set (n = 216)			External validation set (n = 107)		
	Non-recurrence group (n = 393)	Recurrence group (n = 113)	P-value	Non-recurrence group(n = 168)	Recurrence group (n = 48)	P-value	Non-recurrence group (n = 86)	Recurrence group (n = 21)	P-value
General information									
Gender			0.946			0.955			0.439
Female	139 (35)	41 (36)		66 (39)	18 (38)		35 (41)	6 (29)	
Male	254 (65)	72 (64)		102 (61)	30 (62)		51 (59)	15 (71)	
Age(years)	61 (51, 70)	64 (55, 72)	0.058	58.93 ± 13.99	66.1 ± 9.67	**< 0.001**	63.02 ± 12.68	63.14 ± 9.81	0.963
Past medical history									
T2DM			**< 0.001**			**0.002**			0.962
No	239 (61)	40 (35)		107 (64)	18 (38)		42 (49)	11 (52)	
Yes	154 (39)	73 (65)		61 (36)	30 (62)		44 (51)	10 (48)	
Hypertension			0.828			0.152			1
No	288 (73)	81 (72)		125 (74)	30 (62)		48 (56)	12 (57)	
Yes	105 (27)	32 (28)		43 (26)	18 (38)		38 (44)	9 (43)	
Malignant neoplasm			**< 0.001**			**0.002**			**< 0.001**
No	356 (91)	75 (66)		151 (90)	34 (71)		85 (99)	12 (57)	
Yes	37 (9)	38 (34)		17 (10)	14 (29)		1 (1)	9 (43)	
Heart disease			0.399			1			0.516
No	354 (90)	98 (87)		146 (87)	42 (88)		70 (81)	19 (90)	
Yes	39 (10)	15 (13)		22 (13)	6 (12)		16 (19)	2 (10)	
Cerebral infarction			0.388			**0.041**			0.732
No	348 (89)	96 (85)		157 (93)	40 (83)		74 (86)	19 (90)	
Yes	45 (11)	17 (15)		11 (7)	8 (17)		12 (14)	2 (10)	
Biliary disease			**< 0.001**			**< 0.001**			**< 0.001**
No	331 (84)	65 (58)		146 (87)	29 (60)		78 (91)	12 (57)	
Yes	62 (16)	48 (42)		22 (13)	19 (40)		8 (9)	9 (43)	
Laboratory parameters									
ALT (U/L)	51.36 (32.37, 82)	53.78 (32, 77)	0.515	50 (31.96, 83.09)	58.5 (26.66, 79.25)	0.759	51.9 (36.48, 88.9)	45.1 (28.3, 61.8)	0.331
FIB (g/L)	5.9 (4.87, 6.9)	5.47 (4.56, 6.77)	**0.017**	5.64 (4.91, 6.86)	5.56 (4.64, 6.37)	0.343	5.66 (4.85, 6.55)	4.55 (4.01, 5.91)	**0.047**
ALB(g/L)	33 (30, 36.4)	31.9 (28.8, 34.7)	**0.013**	31.05 (28.2, 34.25)	32.25 (30.35, 34.92)	0.198	30.49 ± 5	29.92 ± 5.7	0.676
PLT(×10^9^/L)	217 (120, 310)	193 (110, 285)	0.304	194 (85.5, 290.75)	193.5 (109, 305.25)	0.694	198 (119, 319.25)	202 (123, 324)	0.848
PCT(ng/mL)	2.55 (0.57, 15.27)	22.3 (12.05, 37.65)	**< 0.001**	3.8 (0.83, 20.62)	22.59 (12.07, 43.14)	**< 0.001**	4.3 (3.8, 6.9)	18.82 (11.3, 47.8)	**< 0.001**
CRP (mg/L)	115.89 (61.6, 172.69)	102 (56.81, 139.7)	0.175	114.03 (71.68, 177.77)	104.4 (47.55, 166.3)	0.241	127.65 (127.13, 129.75)	127.65 (127.65, 144.51)	0.738
WBC (×10^9^/L)	9.83 (7.27, 12.79)	8.8 (6.33, 12.6)	0.131	10.39 (7.36, 14.48)	9.98 (7.49, 13.57)	0.677	11.47 ± 3.95	12.17 ± 4.57	0.525
Imaging findings									
Abscess location			0.861			0.786			0.282
Right lobe	266 (68)	79 (70)		115 (68)	35 (73)		50 (58)	9 (43)	
Left lobe	87 (22)	22 (19)		37 (22)	8 (17)		11 (13)	6 (29)	
Left and right lobes	35 (9)	10 (9)		15 (9)	5 (10)		23 (27)	6 (29)	
Caudate lobe	5 (1)	2 (2)		1 (1)	0 (0)		2 (2)	0 (0)	
Number of abscesses			**0.032**			**0.031**			0.524
Single	349 (89)	91 (81)		149 (89)	36 (75)		72 (84)	16 (76)	
Multiple	44 (11)	22 (19)		19 (11)	12 (25)		14 (16)	5 (24)	
Diameter of abscess	58 (40, 78)	60 (46, 81)	0.133	60.49 ± 24.69	55.85 ± 23.62	0.238	56.5 (43.25, 78.75)	56 (49, 66)	0.655
Gas-containing abscess			0.292			0.772			0.135
No	362 (92)	108 (96)		154 (92)	43 (90)		82 (95)	18 (86)	
Yes	31 (8)	5 (4)		14 (8)	5 (10)		4 (5)	3 (14)	
Complications									
Septic shock			**< 0.001**			**< 0.001**			**0.003**
No	364 (93)	79 (70)		154 (92)	33 (69)		84 (98)	16 (76)	
Yes	29 (7)	34 (30)		14 (8)	15 (31)		2 (2)	5 (24)	
Pneumonia			0.46			0.061			0.157
No	331 (84)	99 (88)		152 (90)	38 (79)		65 (76)	12 (57)	
Yes	62 (16)	14 (12)		16 (10)	10 (21)		21 (24)	9 (43)	
Pleural effusion			0.898			0.805			0.354
No	338 (86)	96 (85)		146 (87)	43 (90)		69 (80)	19 (90)	
Yes	55 (14)	17 (15)		22 (13)	5 (10)		17 (20)	2 (10)	
**Treatments**			0.248			1			0.86
Antibiotics	158 (40)	38 (34)		66 (39)	19 (40)		33 (38)	7 (33)	
Antibiotics and surgical drainage	235 (60)	75 (66)		102 (61)	29 (60)		53 (62)	14 (67)	
**SOFA-2 score**	2 (2, 5)	4 (3, 6)	**< 0.001**	3 (2, 5)	5 (4, 7)	**0.005**	2 (1, 3)	6 (4, 8)	**< 0.001**

T2DM, Type 2 Diabetes Mellitus; ALT, Alanine Aminotransferase; FIB, Fibrinogen; ALB, Albumin; PLT, Platelet; PCT, Procalcitonin; CRP, C-reactive Protein; WBC, White Blood Cell; SOFA-2 score, Sequential Organ Failure Assessment 2.0 score.

Bold values denote statistical significance.

**Table 2** summarizes the analysis of relevant variables in the training and external validation sets. The two groups exhibited significant differences in hypertension (P < 0.001), ALB (P < 0.001), CRP (P = 0.019), WBC (P < 0.001), abscess location (P < 0.001), and pneumonia (P = 0.002). **Supplementary Table 1** presents the analysis of relevant variables in the training and testing sets.

**Table 2:** Training set and external validation set variability analysis.

Variables	Total (n = 613)	Training set (n = 506)	External validation set (n = 107)	P-value
General information				
Gender				0.67
Female	221 (36)	180 (36)	41 (38)	
Male	392 (64)	326 (64)	66 (62)	
Age(years)	62 (53, 70)	62 (52, 70)	63 (55, 71)	0.148
Past medical history				
T2DM				0.342
No	332 (54)	279 (55)	53 (50)	
Yes	281 (46)	227 (45)	54 (50)	
Hypertension				**< 0.001**
No	429 (70)	369 (73)	60 (56)	
Yes	184 (30)	137 (27)	47 (44)	
Malignant neoplasm				0.182
No	528 (86)	431 (85)	97 (91)	
Yes	85 (14)	75 (15)	10 (9)	
Heart disease				0.103
No	541 (88)	452 (89)	89 (83)	
Yes	72 (12)	54 (11)	18 (17)	
Cerebral infarction				0.94
No	537 (88)	444 (88)	93 (87)	
Yes	76 (12)	62 (12)	14 (13)	
Biliary disease				0.22
No	486 (79)	396 (78)	90 (84)	
Yes	127 (21)	110 (22)	17 (16)	
Laboratory parameters				
ALT (U/L)	51.7 (32.99, 80)	52 (32, 79.59)	50.1 (35.45, 86.15)	0.635
FIB (g/L)	5.71 (4.7, 6.88)	5.83 (4.74, 6.9)	5.66 (4.62, 6.36)	0.257
ALB(g/L)	32.53 ± 5.11	32.98 ± 4.99	30.38 ± 5.12	**< 0.001**
PLT(×10^9^/L)	211 (116, 308)	212.5 (115.25, 304.25)	199 (120, 321.5)	0.752
PCT(ng/mL)	4.65 (1.14, 21.48)	5.7 (0.9, 22.43)	4.5 (3.8, 13)	0.259
CRP (mg/L)	123 (69.8, 164.12)	110 (60.92, 168.57)	127.65 (127.28, 132)	**0.019**
WBC (×10^9^/L)	10.06 (7.25, 13.08)	9.67 (6.86, 12.71)	11.63 (8.87, 14.06)	**< 0.001**
Imaging findings				
Abscess location				**< 0.001**
Right lobe	404 (66)	345 (68)	59 (55)	
Left lobe	126 (21)	109 (22)	17 (16)	
Left and right lobes	74 (12)	45 (9)	29 (27)	
Caudate lobe	9 (1)	7 (1)	2 (2)	
Number of abscesses				0.259
Single	528 (86)	440 (87)	88 (82)	
Multiple	85 (14)	66 (13)	19 (18)	
Diameter of abscess	58 (41, 79)	58 (41, 78.75)	56 (44.5, 78.5)	0.811
Gas-containing abscess				0.998
No	570 (93)	470 (93)	100 (93)	
Yes	43 (7)	36 (7)	7 (7)	
Complications				
Septic shock				0.114
No	543 (89)	443 (88)	100 (93)	
Yes	70 (11)	63 (12)	7 (7)	
Pneumonia				**0.002**
No	507 (83)	430 (85)	77 (72)	
Yes	106 (17)	76 (15)	30 (28)	
Pleural effusion				0.434
No	522 (85)	434 (86)	88 (82)	
Yes	91 (15)	72 (14)	19 (18)	
**Treatments**				0.879
Antibiotics	236 (38)	196 (39)	40 (37)	
Antibiotics and surgical drainage	377 (62)	310 (61)	67 (63)	
**SOFA-2 score**	3 (2, 5)	3 (2, 5)	3 (2, 4)	0.267

T2DM, Type 2 Diabetes Mellitus; ALT, Alanine Aminotransferase; FIB, Fibrinogen; ALB, Albumin; PLT, Platelet; PCT, Procalcitonin; CRP, C-reactive Protein; WBC, White Blood Cell; SOFA-2 score, Sequential Organ Failure Assessment 2.0 score.

Bold values denote statistical significance.

### 3.2 Development of the model

**Figure 2:**
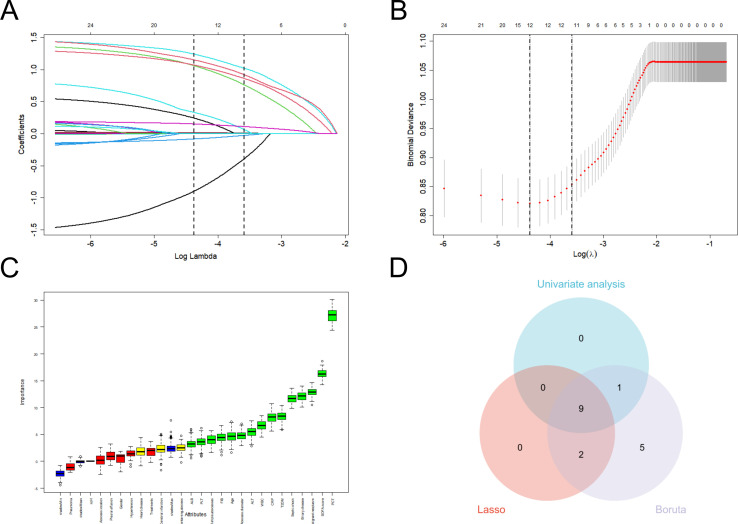
Features selected by Lasso, Boruta and univariate analysis. **(A)** The Lasso regression coefficient profiles of all baseline characteristics. **(B)** The optimal lambda selection in the Lassoregression with 10-fold cross-validation. Misclassification errors of different variables against log(lambda) are revealed. The two vertical dashed lines represent the optimal value under the minimum criterion and 1-SE criterion, respectively. The “lambda” is the tuning parameter. A total of 11 predictors with non-zero coefficients are identified. **(C)** Variables selected by Boruta algorithm. The minimum, average and maximum shadow score are shown in blue. In terms of the score of feature importance, the 17 variables in green are regarded as important variables, while yellow are neutral and red are rejected. **(D)**The Venn diagram of features elected by Boruta, Lasso and univariate analysis. The intersection results of three methods yield 9 clinical characteristics. SE, standard error; Lasso, least absolute shrinkage and selection operator.

As shown in **Supplementary Table 2**, univariate analysis revealed 10 variables showing significant differences between the recurrent and non-recurrent groups in the training set (P < 0.05). From the baseline characteristics, LASSO regression selected 11 variables as potential predictors (**Figures 2A, B**). The Boruta algorithm also effectively selected 17 potential predictors based on the Z-values (importance) (**Figure 2C**). **Supplementary Table 3** shows the variables identified by the Boruta algorithm and LASSO regression. To derive optimal features for the model, the results of these three methods were intersected, which yielded 9 critical clinical characteristics, including age, T2DM, malignant neoplasm, biliary disease, FIB, PCT, multiple abscesses, septic shock, and SOFA-2 scores; these clinical characteristics then served as key predictors to establish ML-based prediction models (**Figure 2D**). A comparison between the model constructed using 9 screened features and models with other feature sets is shown in **Supplementary Table 4**.

### 3.3 Evaluation of the models

The models’ performance was evaluated in the training, testing, and external validation sets (**Table 3**). In the training set, the optimized XGBoost model demonstrated excellent comprehensive performance, with an AUC of 0.936 (95% CI: 0.914–0.959) and accuracy of 0.852 (95% CI: 0.820–0.879). It also exhibited satisfactory sensitivity (0.876), specificity (0.845), precision (PPV) (0.619), F1 score (0.725), and NPV (0.960). Although LightGBM and CatBoost showed nearly perfect performance in the training set, their results indicated obvious overfitting. However, in five-fold cross-validation, the XGBoost model showed highly stable performance, with a mean AUC of 0.889 (Standard Deviation = 0.014), indicating strong robustness and favorable generalizability. The results of cross-validation are presented in **Supplementary Table 5** and **Supplementary Figure 4**.

**Table 3:** Comparative analysis of the performance outcomes across seven machine learning models.

	Model	AUC(95%CI)	Accuracy	F1 score	Sensitivity	Specificity	Brier score	Precision (PPV)	NPV
Training Set	LM	0.847(0.807-0.887)	0.771	0.618	0.832	0.753	0.122	0.492	0.940
	DT	0.879(0.840-0.918)	0.767	0.622	0.858	0.741	0.095	0.487	0.948
	RF	0.984(0.976-0.992)	0.919	0.841	0.956	0.908	0.052	0.750	0.986
	XGBoost	0.936(0.914-0.959)	0.852	0.725	0.876	0.845	0.085	0.619	0.960
	LightGBM	1.000(1.000-1.000)	1.000	1.000	1.000	1.000	0.006	1.000	1.000
	CatBoost	1.000(1.000-1.000)	1.000	1.000	1.000	1.000	0.004	1.000	1.000
	MLP	0.885(0.851-0.919)	0.804	0.653	0.823	0.800	0.147	0.541	0.940
Testing Set	LM	0.820(0.743-0.897)	0.741	0.563	0.750	0.738	0.120	0.450	0.912
	DT	0.823(0.754-0.891)	0.694	0.542	0.813	0.661	0.142	0.406	0.925
	RF	0.887(0.818-0.956)	0.815	0.667	0.833	0.810	0.089	0.556	0.944
	XGBoost	0.868(0.799-0.938)	0.810	0.661	0.833	0.804	0.102	0.548	0.944
	LightGBM	0.907(0.848-0.967)	0.912	0.782	0.708	0.970	0.077	0.872	0.921
	CatBoost	0.895(0.841-0.950)	0.870	0.696	0.667	0.929	0.100	0.727	0.907
	MLP	0.837(0.768-0.906)	0.801	0.626	0.750	0.816	0.154	0.537	0.920
External Validation Set	LM	0.813(0.687-0.939)	0.794	0.577	0.714	0.814	0.107	0.484	0.921
	DT	0.873(0.774-0.972)	0.822	0.642	0.810	0.826	0.098	0.531	0.947
	RF	0.900(0.812-0.988)	0.841	0.638	0.714	0.872	0.084	0.577	0.926
	XGBoost	0.904(0.819-0.988)	0.851	0.667	0.762	0.872	0.089	0.593	0.938
	LightGBM	0.831(0.743-0.919)	0.832	0.438	0.333	0.954	0.126	0.636	0.854
	CatBoost	0.782(0.664-0.901)	0.879	0.606	0.476	0.977	0.114	0.833	0.884
	MLP	0.840(0.737-0.943)	0.813	0.583	0.667	0.849	0.146	0.519	0.913

LM, logistic regression model; DT, decision tree; RF, random forest; XGBoost, eXtreme gradient boosting; LightGBM, light gradient boosting machine; CatBoost, categorical boosting; MLP, multilayer perceptron; AUC, area under the receiver operating characteristic curve; PPV, Positive Predictive Value; NPV, Negative Predictive Value.

**Figure 3:**
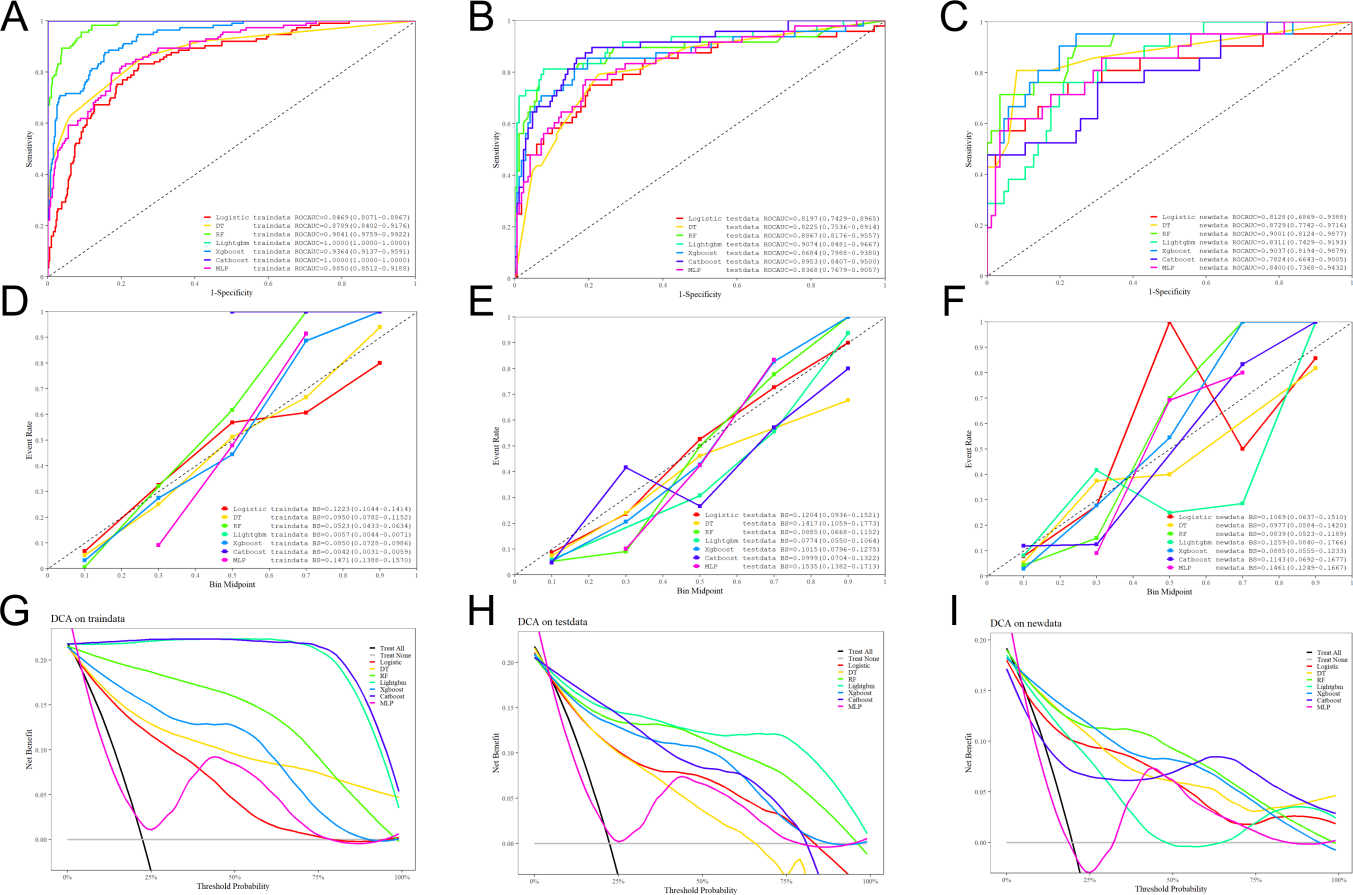
The performance and comparison of seven different predictive models. **(A)** ROC Curves for the training set; **(B)** ROC Curves for the test set; **(C)** ROC Curves for the external validation set; **(D)** Calibration Curve for the training set; **(E)** Calibration Curve for the test set; **(F)** Calibration Curve for the external validation set; **(G)** Decision Curve Analysis for the training set; **(H)** Decision Curve Analysis for the test set; **(I)** Decision Curve Analysis for the external validation set.

In the testing set, the optimized XGBoost model maintained good stability, with an AUC of 0.868 (95% CI: 0.799–0.938) and accuracy of 0.810 (95% CI: 0.757–0.857). Its sensitivity, specificity, precision, F1 score, and NPV were 0.833, 0.804, 0.548, 0.661, and 0.944, respectively. The overall performance was better than that of the logistic regression, decision tree, and MLP models, and more stable than the RF model. CC analysis and DCA further verified the reliable predictive ability and clinical applicability of the XGBoost model in the testing set (**Figure 3**).

In the external validation set, the optimized XGBoost model achieved the highest AUC of 0.904 (95% CI: 0.819–0.988) among all models, with an accuracy of 0.851 (95% CI: 0.776–0.916), sensitivity of 0.762, specificity of 0.872, precision (PPV) of 0.593, F1 score of 0.667, and NPV of 0.938. The Brier score was 0.089, indicating good calibration. DCA demonstrated that the XGBoost model achieved positive net benefit across almost the entire clinically relevant threshold range (0–100%). The decision curve of the model was consistently higher than both the “treat all” and “treat none” strategies, suggesting superior clinical utility. Although the RF model showed favorable performance in the training and internal testing sets, it had a lower external validation AUC (0.900 vs. 0.904) and weaker generalization than the optimized XGBoost model. Meanwhile, LightGBM and CatBoost suffered from severe overfitting, with sharply decreased performance in external validation. In contrast, the XGBoost model maintained balanced and stable performance across all datasets, showing stronger robustness and clinical applicability. Therefore, XGBoost was selected as the final optimal model.

**Figure 4:**
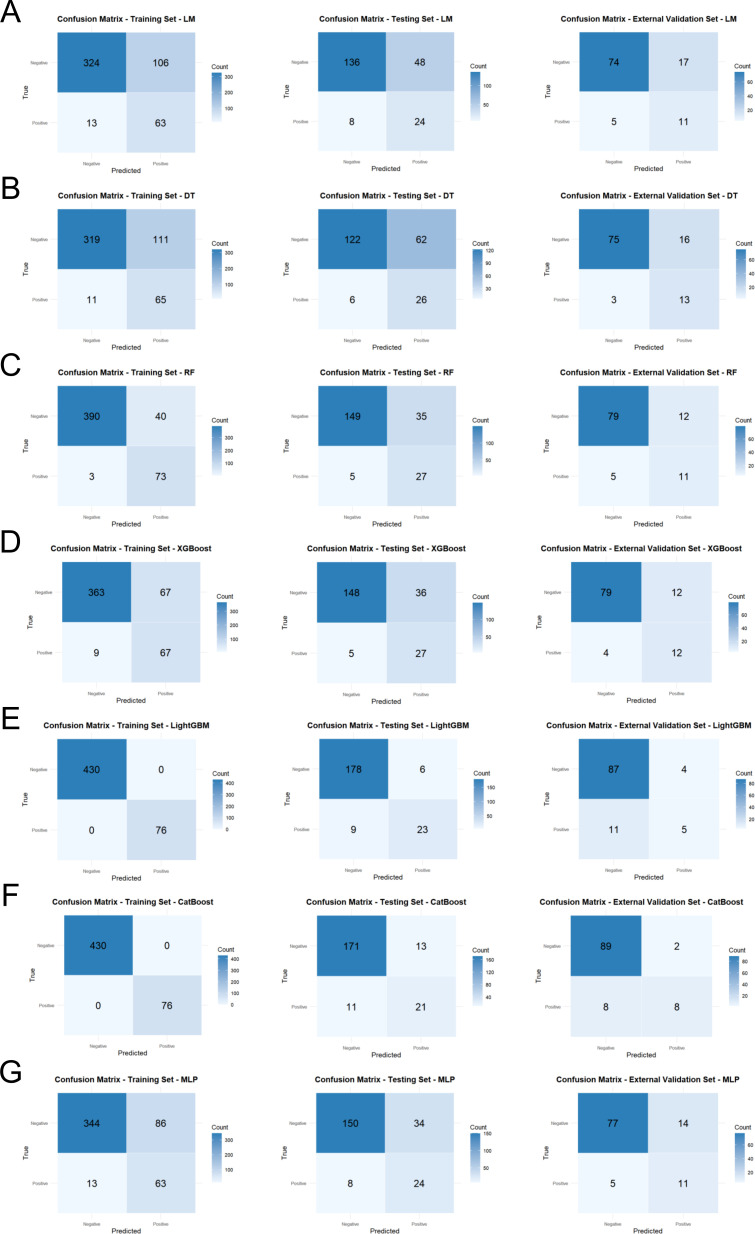
Confusion matrices of seven machine learning models across three datasets. **(A-G)** correspond to the following models: **(A)** LM, **(B)** DT, **(C)** RF, **(D)** XGBoost, **(E)** LightGBM, **(F)** CatBoost, and **(G)** MLP. Each panel presents true positives (TP), false positives (FP), true negatives (TN), and false negatives (FN), enabling direct comparison of classification accuracy and error distribution across models. LM, logistic regression model; DT, decision tree; RF, random forest; XGBoost, eXtreme gradient boosting; LightGBM, light gradient boosting machine; CatBoost, categorical boosting; MLP, multilayer perceptron.

The confusion matrix analysis also supported the generalizability of the XGBoost model. In both testing and external validation sets, the XGBoost classifier achieved a high number of true positives and true negatives with relatively few misclassifications (**Figure 4D**). In contrast, the RF and LightGBM models (**Figures 4C, E**) yielded more false positives in both testing and external validation sets, consistent with their weaker generalization. In addition, the precision-recall curves of the training set, testing set and external validation set are presented in **Supplementary Figure 3**.

### 3.4 Model interpretability

**Figure 5:**
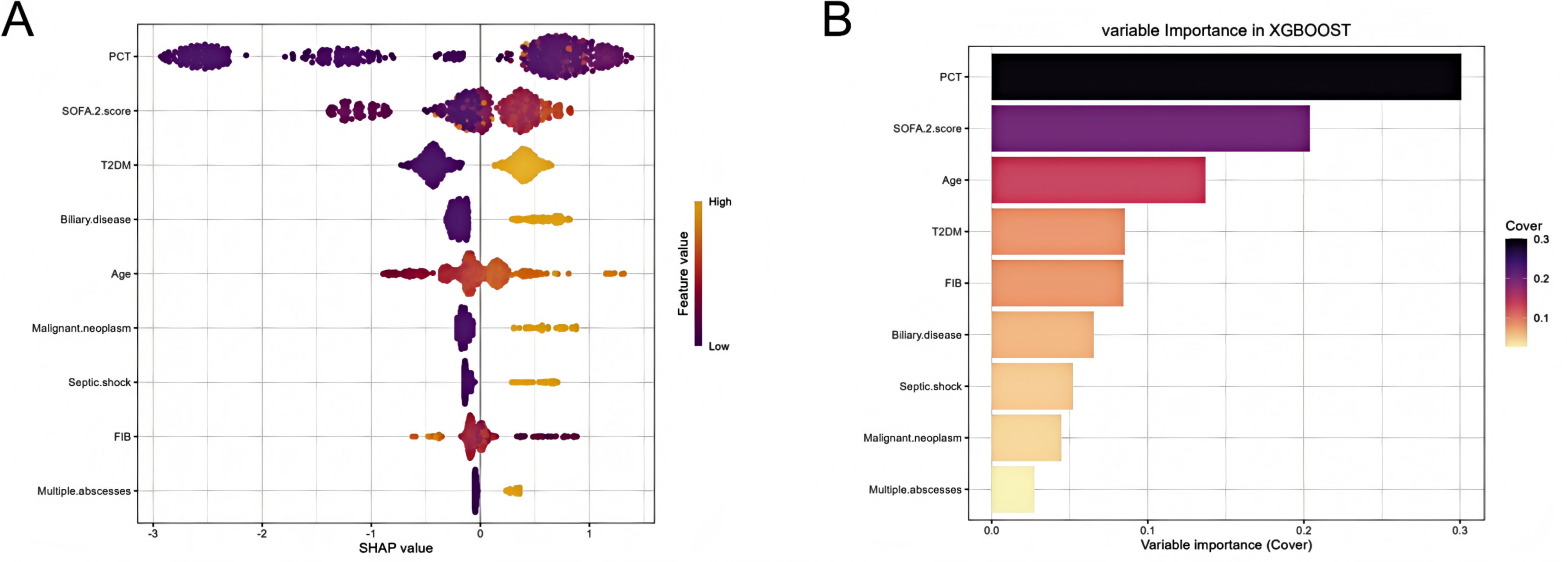
SHAP summary plot and feature importance of the XGBoost model. **(A)** SHAP summary plot shows feature importance for each predictor of the XGBoost model in descending order. The upper predictors are more important to the model’s predictive outcome. A dot is created for each feature attribution value for the XGBoost model of each patient. The further away a dot is from the baseline SHAP value of zero, the stronger it effects the model output. Dots are colored according to the values of features. Yellow represents higher feature values and purple represents lower feature values. **(B)** Variable importance plot (Cover metric) of the XGBoost model in descending order. T2DM, type 2 diabetes mellitus; FIB, fibrinogen; PCT, procalcitonin; SOFA-2 score, sequential organ failure assessment 2.0 score. SHAP, SHapley Additive exPlanations.

The SHAP values were estimated to clarify how the XGBoost model predicted outcomes. **Figure 5A** illustrates a SHAP summary plot, showing the SHAP value distribution of clinical features (e.g., PCT and SOFA-2 score). The colors identify the feature values (orange = high, purple = low), indicating the direction and extent of features’ impact on model predictions. **Figure 5B** is the variable importance plot (cover metric) of the XGBoost model, indicating that the PCT feature contributes most to model decisions.

**Figure 6:**
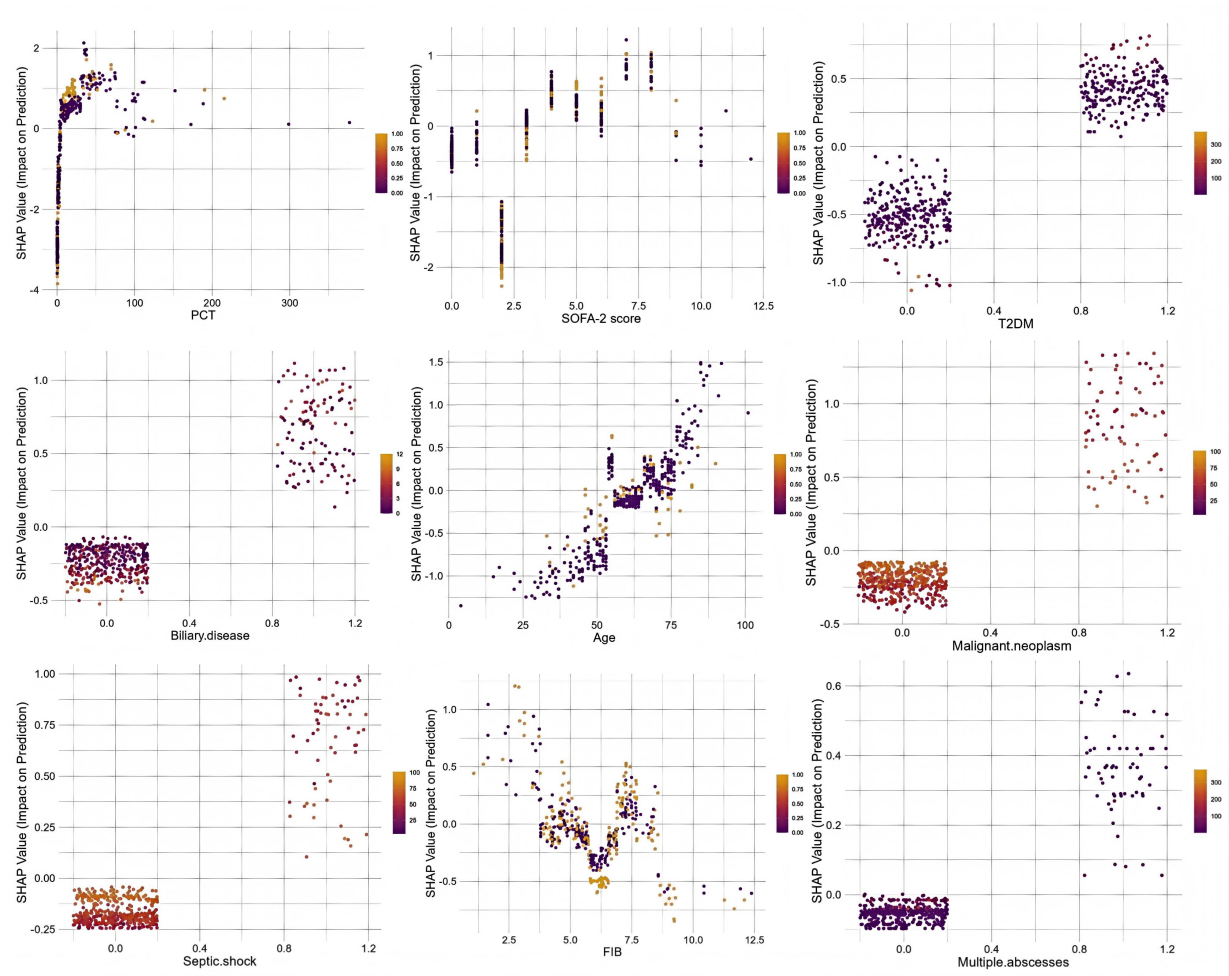
SHAP dependence plot. Each dependence plot shows how a single feature affects the output of the prediction model, and each dot represents a single child. SHAP values are represented by the y-axis, and actual values are represented by the x-axis. The SHAP values for specific features exceeding zero push the decision toward the ‘‘Recurrence” class.T2DM, type 2 diabetes mellitus; FIB, fibrinogen; PCT, procalcitonin; SOFA-2 score, sequential organ failure assessment 2.0 score; SHAP, SHapley Additive exPlanations.

Next, we quantitatively visualized the association between the major risk factors and clinical outcomes. SHAP dependence plots (**Figure 6**) delineated the quantitative associations between candidate predictors and KPLA recurrence risk. PCT levels (>2.0 ng/mL) were accompanied by a rapid increase in SHAP values. A SOFA-2 score exceeding 5 correlated with a marked upward shift in SHAP values. The FIB level exhibited a nonlinear relationship with recurrence risk, as SHAP values initially increased and subsequently decreased with increasing FIB levels. Malignant neoplasm and septic shock showed strong positive correlations with recurrence risk based on SHAP contribution values. SHAP values were also associated with advanced age, T2DM, and biliary disease.

**Figure 7:**
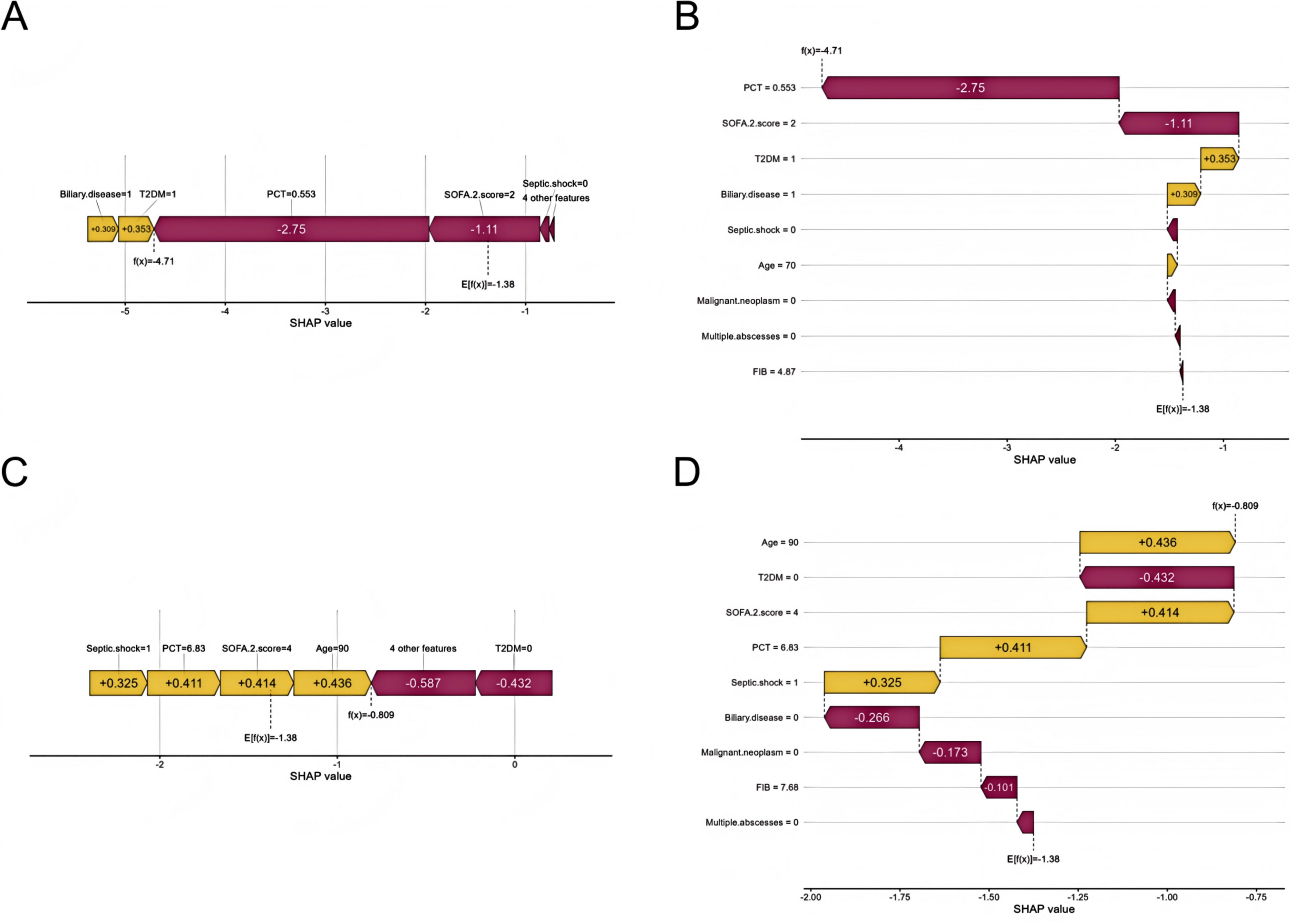
SHAP individual prediction visualizations plots. yellow bars represent positive contributions and purple bars represent negative contributions. **(A)** SHAP force plot for an individual patient with recurrent KPLA; **(B)** SHAP waterfall plot for an individual patient with recurrent KPLA; **(C)** SHAP force plot for an individual patient with non-recurrent KPLA; **(D)** SHAP waterfall plot for an individual patient with non-recurrent KPLA. E[f(x)] denotes the baseline predicted value, while f(x) represents the final predicted value. T2DM, type 2 diabetes mellitus; FIB, fibrinogen; PCT, procalcitonin; SOFA-2 score, sequential organ failure assessment 2.0 score; SHAP, SHapley Additive exPlanations.

**Figures 7A, C** are force plots illustrating how each feature contributes to the final predicted value f(X) starting from the model’s baseline prediction value (base value). Purple and yellow arrows represent factors that decrease and increase the predicted value, respectively. **Figures 7B, D** depict waterfall plots showing the contribution of different features to the model’s prediction output (SHAP values) for a given patient. Each line represents a feature: purple indicates a negative contribution to the prediction result, while yellow indicates a positive contribution.

**Figures 7A, B** present results for one individual in the non-recurrence group. PCT and SOFA-2 score were the main features with negative contributions to the model’s predicted value, and the final predicted value was −4.71. T2DM and biliary disease showed positive contributions to the predicted value. **Figures 7C, D** present results for another individual in the recurrence group. Age, SOFA-2 score, and PCT were the main features with positive contributions to the model’s predicted value, and the final predicted value was 0.809.

### 3.5 Implementation of the web-based tool

**Figure 8:**
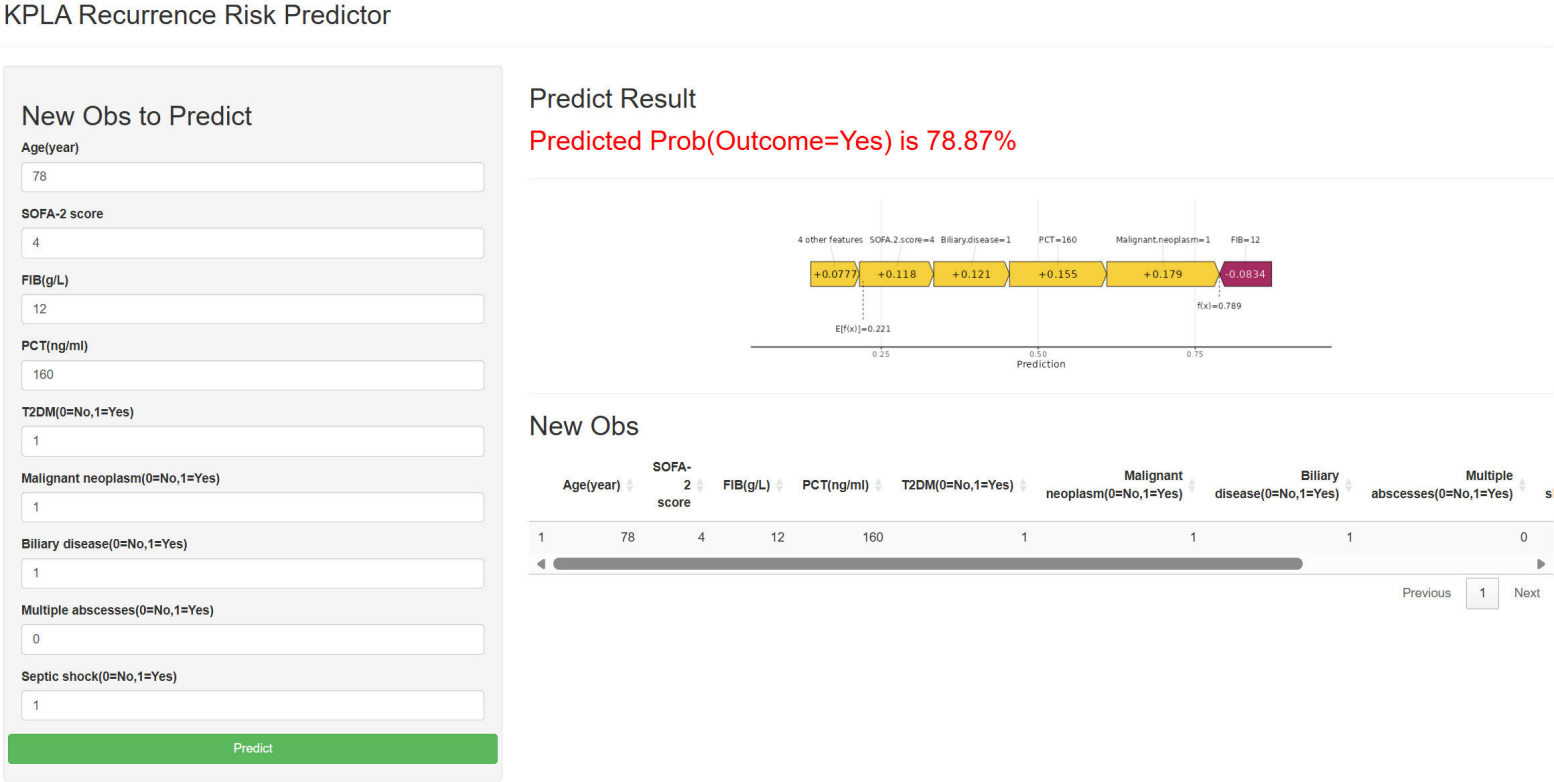
A web-based calculator for predicting recurrence of KPLA patients. KPLA, Klebsiella pneumoniae liver abscess; T2DM, type 2 diabetes mellitus; FIB, fibrinogen; PCT, procalcitonin; SOFA-2 score, sequential organ failure assessment 2.0 score; SHAP, SHapley Additive exPlanations.

The final XGBoost model was deployed as an interactive web-based risk prediction tool (**Figure 8**). The tool interface requires data entry of the 9 key variables: age, SOFA-2 score, FIB, PCT, T2DM, malignant neoplasm, biliary disease, multiple abscesses, and septic shock. Subsequently, the tool generates an individualized prediction of short-term functional outcomes at discharge, accompanied by graphical explanations based on the model’s logic. By utilizing the R package “shiny” (Xiao and Lam, 2022), a web-based application (https://kplariskpredict.shinyapps.io/xgboost/) was developed to facilitate online accessibility of the predictive model. This tool offers real-time interaction, provides remote access, and can be integrated into future clinical decision support systems.

## 4 Discussion

By using real-world EMR data from three tertiary hospitals, this study evaluated seven ML algorithms to construct a recurrence risk prediction model for KPLA. This model was further validated in a quasi-external cohort to confirm its robustness and generalizability. Model interpretability was enhanced by applying SHAP analysis, and an interactive web-based tool was developed to provide reliable support to clinicians to identify patients at a high risk of KPLA recurrence and formulate customized intervention plans (Lin et al., 2024; Yan et al., 2024; Yuan et al., 2025). For high-risk patients, clinicians can optimize antibiotic use based on susceptibility results, perform drainage according to guideline indications, and strengthen follow-up management, rather than blindly extending the course or enhancing drainage. These findings support the use of this model for individualized recurrence risk stratification and precise clinical decision-making in patients with KPLA.

Compared with single-center studies with small sample sizes (Zhang et al., 2025b), this study adopted multicenter real-world data, which reflect a wider spectrum of patient characteristics and clinical management practices. The XGBoost model, based on a gradient boosting framework with regularization optimization, can effectively capture the complex nonlinear relationships among clinical indicators (Li et al., 2026), which is well-matched with the multifactorial pathogenesis of KPLA recurrence (including host immunity, pathogen invasion, and treatment response). In this multicenter high-dimensional dataset, the XGBoost model exhibited better performance in fitting complex nonlinear associations than traditional logistic regression (which can address nonlinear features via polynomial transformation or LASSO regularization). Furthermore, the XGBoost model has strong robustness in processing missing values, outliers (Zhang et al., 2020), and high-dimensional features in multicenter clinical data, which markedly improves its applicability in real-world clinical settings. Compared with the conventional logistic regression model, the XGBoost algorithm exhibited better discriminative ability and predictive stability in both internal and external validation, with prominent advantages in sensitivity, negative predictive value and overall comprehensive performance.

The mechanisms underlying the association between the identified nine core predictive indicators and KPLA recurrence were supported by clinical evidence. The SHAP analysis further clarified the weightage of influence of each indicator. As a sensitive marker of bacterial infection, an elevated PCT level not only reflects the pathogen load and inflammatory response intensity but also indicates increased difficulty in controlling the infection; this aspect is consistent with previous findings that patients with a persistently abnormal PCT level have a significantly higher risk of infection recurrence (Stalenhoef et al., 2019; Mućka et al., 2025). The SOFA-2 score is a quantitative indicator of organ function damage, and its elevation implies a decrease in the patient’s overall compensatory capacity (Ranzani et al., 2025). A high SOFA-2 score also indicates that, following infection, patients are more likely to progress to severe illness and recover slowly, which could play a critical role in inducing recurrence (Lambden et al., 2019). T2DM and malignant neoplasm indirectly increase the recurrence risk by impairing the immune function of the patient. Long-term hyperglycemia in diabetic patients suppresses the phagocytic ability of neutrophils and the activity of lymphocytes, leading to incomplete clearance of infection (Cilloniz and Torres, 2024). Patients with malignant tumors exhibit immunosuppression due to chemotherapy and radiotherapy, together with other health issues such as malnutrition and insufficient organ function reserve, all of which create favorable conditions for pathogen re-colonization (Tang et al., 2020).

Biliary disease, a common factor promoting KPLA, causes cholestasis and biliary obstruction, which destroy the local microenvironment and diminish the effects of anti-infection treatment (Ren et al., 2020). Consequently, abscess lesions are incompletely resolved and pose as a hidden risk for recurrence. Additionally, the natural decline of immune function in elderly patients leads to reduced pathogen clearance efficiency. Even if an elderly patient achieves clinical remission through initial treatment, residual pathogens can re-colonize due to a lower immunity level and cause infection recurrence (Yoshikawa et al., 2020; Li et al., 2022). Moreover, elderly patients frequently have multiple comorbidities, resulting in insufficient microcirculatory perfusion of liver tissues and weakened repair capacity (Li et al., 2022). Multiple abscesses often indicate stronger pathogen invasiveness and a wider range of infection spread. Occult microabscesses might also exist, which are prone to residual infection development due to insufficient drug penetration or incomplete drainage during treatment (Niederreiter et al., 2025), thereby increasing recurrence risk. The bidirectional association of FIB with KPLA recurrence risk may be associated with its dual roles in infection and coagulation. This conclusion is a clinical inference and needs to be verified by further basic research on the specific molecular mechanism. A history of septic shock indicates that the patient has experienced a severe infection attack, with impaired immune memory and tissue repair ability, further increasing the susceptibility to recurrence (Tang et al., 2023). In addition, blood glucose, blood urea nitrogen, and uric acid were excluded due to a missing rate exceeding 20% to avoid imputation bias. Blood glucose reflects glycemic control and immune function status; blood urea nitrogen reflects renal function and antibiotic efficacy; uric acid is associated with hepatic inflammatory status. Although these variables are related to KPLA recurrence, they were ultimately not included in the analysis due to data quality limitations. Furthermore, pathogen-related factors, including virulence characteristics and antimicrobial resistance profiles, may also contribute to KPLA recurrence. Future studies incorporating these microbiological indicators are warranted to further improve the performance and clinical applicability of the prediction model.

Considering the limitations of “black box” ML models in clinical practice, SHAP analysis was applied to clarify the global and individual contributions of the predictors included in the XGBoost model. Summary plots showed that PCT, SOFA-2 score, and T2DM were the most influential predictors. Although T2DM and biliary disease were identified as risk factors for KPLA recurrence, their SHAP contribution values were lower than those of PCT and SOFA-2 score. This might be attributed to the binary definition of T2DM lacking glycemic control information and the non-specific nature of biliary disease for KPLA. The above results reflect statistical associations between features and KPLA recurrence risk, rather than definitive causal relationships. We avoided over-interpretation of the model outputs and strictly distinguished statistical significance from clinical causal plausibility to ensure the rationality of result interpretation. Waterfall plots and force plots provided case-level explanations of how individual predictors shaped outcome estimates. These visualization methods may improve our understanding of model predictions and support their clinical application. We developed a publicly accessible online risk tool for visualizing prediction results. In the future, this tool can be further integrated into hospital EMR systems for automatic patient data extraction and real-time risk alerts, while providing data support for clinical pathway development and optimal allocation of medical insurance resources. The proposed model and the accompanying web-based tool represent a promising proof-of-concept for risk stratification. However, they require prospective validation in diverse clinical settings, formal user testing with clinicians, and evaluation of their impact on clinical decision-making and patient outcomes before they can be recommended for routine clinical use.

This study proposes a full-chain “algorithm-indicator-tool” system as an exploratory translational framework for bridging basic research and clinical practice. It presents three potential clinical values: First, standardized risk assessment — the 9 core predictors are all routine clinical indicators, which avoids additional medical costs and converts KPLA recurrence risk evaluation from subjective empirical judgment to an objective data-driven process, making it applicable for medical institutions at all levels including primary care settings. Second, individualized intervention guidance — the web-based tool supports real-time risk stratification at initial diagnosis. For high-risk patients (e.g., PCT > 2.0 ng/mL, SOFA-2 score > 5, or with malignant neoplasm), it assists clinicians in implementing guideline-directed standardized treatment and intensive follow-up management, thereby reducing overtreatment in low-risk patients. Third, clinical management optimization — the stable performance validated in the multicenter cohort supports its regional clinical application, and further multi-regional external validation is warranted before wider promotion. Its compatibility with electronic medical record systems enables automated data extraction and real-time risk calculation, providing evidence support for the development of clinical pathways and precise allocation of medical resources. This framework also offers a reproducible paradigm for precision management of infectious liver diseases, facilitating the shift of care from empirical treatment toward individualized precision management.

Although the present study has several strengths, it still has some inevitable limitations. First, this was a multicenter retrospective study with only 12 months of follow-up, which may introduce inherent selection bias and information bias. Furthermore, we only assessed recurrence within 1 year without collecting the specific time to recurrence; future time-to-event studies are warranted to clarify the time-dependent pattern of KPLA recurrence. Second, due to the high missing rate of relevant data, HbA1c and continuous glycemic indicators were not included in the analysis; T2DM was only incorporated as a binary variable, which may underestimate the actual impact of glycemic control on disease recurrence. Future research will adopt advanced missing data imputation methods to compensate for incomplete variable collection and improve analytical reliability. Third, this study lacked detailed microbiological data, such as virulence genes and resistance profiles. These data are critical for clarifying recurrence mechanisms and optimizing antibiotic therapy. Future models combining pathogen information will be more comprehensive. Fourth, all enrolled participants were recruited from northern China, and quasi-external validation was conducted within the same medical system, which limits the cross-regional generalizability of our model. Fifth, the relatively high AUC value may be partially affected by population homogeneity, and validation across different departments may bring potential selection bias. Finally, the online prediction tool lacks prospective clinical validation. Further well-designed prospective studies with complete glycemic and antimicrobial susceptibility data, as well as multi-center and cross-regional verification, are needed to further optimize and generalize the model.

## 5 Conclusion

Here, we developed an ML-based model trained on the EMR data of KPLA patients from two hospitals and validated it in an additional quasi-external cohort from a third hospital to predict KPLA recurrence. The constructed model exhibited good predictive accuracy and calibration. Our study highlights the value of combining clinical features and laboratory indicators to predict KPLA recurrence. Moreover, the selected variables proved useful to predict recurrence risk in KPLA patients. Future studies should expand the cohort size, validate the model in diverse clinical settings, and integrate dynamic variables and longitudinal follow-up to enhance the model’s clinical applicability and generalizability.

## Data Availability

The datasets analyzed during the current study are available from the corresponding author upon reasonable request. Requests to access these datasets should be directed to AY, ccw1979@126.com.
