# Supplementary material for: A machine learning-based online prediction model for recurrence risk in patients with Klebsiella pneumoniae liver abscess: a multicenter retrospective study
Source: Front Cell Infect Microbiol. 2026 May 20;16:1830022. doi: 10.3389/fcimb.2026.1830022 (PMC13229994; doi:10.3389/fcimb.2026.1830022)
Supplement: Supplementary file 1 [file Table1.docx]

Supplementary Material

**
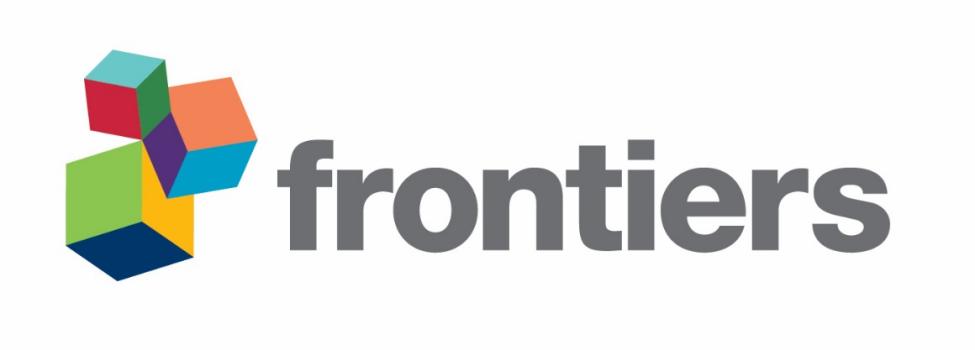
**

**Supplementary Table 1. Training Set and Testing Set variability analysis.**

| **Variables** | **Total (n = 722)** | **Training Set**  **(n = 506)** | **Testing Set (n = 216)** | **P-value** |
| --- | --- | --- | --- | --- |
| **General information** | | | | |
| Gender |  |  |  | 0.446 |
| Female | 264 (37) | 180 (36) | 84 (39) |  |
| Male | 458 (63) | 326 (64) | 132 (61) |  |
| Age(years) | 61 (52, 70) | 62 (52, 70) | 60.5 (52.75, 70) | 0.655 |
| **Past medical history** | | | | |
| T2DM |  |  |  | 0.552 |
| No | 404 (56) | 279 (55) | 125 (58) |  |
| Yes | 318 (44) | 227 (45) | 91 (42) |  |
| Hypertension |  |  |  | 0.818 |
| No | 524 (73) | 369 (73) | 155 (72) |  |
| Yes | 198 (27) | 137 (27) | 61 (28) |  |
| Malignant neoplasm |  |  |  | 0.961 |
| No | 616 (85) | 431 (85) | 185 (86) |  |
| Yes | 106 (15) | 75 (15) | 31 (14) |  |
| Heart disease |  |  |  | 0.447 |
| No | 640 (89) | 452 (89) | 188 (87) |  |
| Yes | 82 (11) | 54 (11) | 28 (13) |  |
| Cerebral infarction |  |  |  | 0.223 |
| No | 641 (89) | 444 (88) | 197 (91) |  |
| Yes | 81 (11) | 62 (12) | 19 (9) |  |
| Biliary disease |  |  |  | 0.463 |
| No | 571 (79) | 396 (78) | 175 (81) |  |
| Yes | 151 (21) | 110 (22) | 41 (19) |  |
| **Laboratory parameters** | | | | |
| ALT (U/L) | 51.89 (32, 80) | 52 (32, 79.59) | 51.09 (31.03, 80.25) | 0.982 |
| FIB (g/L) | 5.73 (4.77, 6.88) | 5.83 (4.74, 6.9) | 5.64 (4.81, 6.67) | 0.354 |
| ALB(g/L) | 32.4 (29.1, 35.8) | 32.72 (29.55, 36) | 31.57 (28.41, 34.42) | < 0.001 |
| PLT(×10^9^/L) | 208.5 (110, 299.75) | 212.5 (115.25, 304.25) | 194 (91.75, 295) | 0.131 |
| PCT(ng/mL) | 6.41 (0.97, 23.98) | 5.7 (0.9, 22.43) | 8.38 (1.01, 30.54) | 0.255 |
| CRP (mg/L) | 111.6 (61.6, 169.57) | 110 (60.92, 168.57) | 111.99 (66.25, 172.44) | 0.595 |
| WBC (×10^9^/L) | 9.91 (7.06, 13.05) | 9.67 (6.86, 12.71) | 10.27 (7.4, 14.09) | 0.053 |
| **Imaging findings** | | | | |
| Abscess location |  |  |  | 0.825 |
| Right lobe | 495 (69) | 345 (68) | 150 (69) |  |
| Left lobe | 154 (21) | 109 (22) | 45 (21) |  |
| Left and right lobes | 65 (9) | 45 (9) | 20 (9) |  |
| Caudate lobe | 8 (1) | 7 (1) | 1 (0) |  |
| Number of abscesses |  |  |  | 0.724 |
| Single | 625 (87) | 440 (87) | 185 (86) |  |
| Multiple | 97 (13) | 66 (13) | 31 (14) |  |
| Diameter of abscess | 58 (41, 78) | 58 (41, 78.75) | 58 (41.5, 75.25) | 0.693 |
| Gas-containing abscess |  |  |  | 0.531 |
| No | 667 (92) | 470 (93) | 197 (91) |  |
| Yes | 55 (8) | 36 (7) | 19 (9) |  |
| **Complications** | | | | |
| Septic shock |  |  |  | 0.812 |
| No | 630 (87) | 443 (88) | 187 (87) |  |
| Yes | 92 (13) | 63 (12) | 29 (13) |  |
| Pneumonia |  |  |  | 0.349 |
| No | 620 (86) | 430 (85) | 190 (88) |  |
| Yes | 102 (14) | 76 (15) | 26 (12) |  |
| Pleural effusion |  |  |  | 0.617 |
| No | 623 (86) | 434 (86) | 189 (88) |  |
| Yes | 99 (14) | 72 (14) | 27 (12) |  |
| **Treatments** |  |  |  | 0.942 |
| Antibiotics | 281 (39) | 196 (39) | 85 (39) |  |
| Antibiotics and surgical drainage | 441 (61) | 310 (61) | 131 (61) |  |
| **SOFA-2 score** | 3 (2, 5) | 3 (2, 5) | 4 (2, 6) | < 0.001 |

T2DM:Type 2 Diabetes Mellitus;ALT:Alanine Aminotransferase;FIB:Fibrinogen;ALB:Albumin;PLT:Platelet;PCT:Procalcitonin;

CRP:C-reactive Protein;WBC:White Blood Cell;SOFA-2 score:Sequential Organ Failure Assessment 2.0 score.

**Supplementary Table 2. Univariate logistic regression analyse.**

| **characteristics** | **Uni-B** | **Uni-SE** | **Uning-OR** | **Uni-CI** | **Uni-Z** | **Uni-P** |
| --- | --- | --- | --- | --- | --- | --- |
| Gender | -0.04 | 0.222 | 0.961 | 0.961(0.622-1.485) | -0.179 | 0.858 |
| Age | 0.02 | 0.008 | 1.02 | 1.02(1.004-1.036) | 2.417 | **0.016** |
| T2DM | 1.041 | 0.222 | 2.832 | 2.832(1.833-4.376) | 4.685 | **<0.001** |
| Hypertension | 0.08 | 0.238 | 1.084 | 1.084(0.68-1.728) | 0.337 | 0.736 |
| Malignant neoplasm | 1.584 | 0.264 | 4.875 | 4.875(2.906-8.179) | 6.01 | **<0.001** |
| Heart disease | 0.329 | 0.325 | 1.389 | 1.389(0.735-2.627) | 1.013 | 0.311 |
| Cerebral infarction | 0.314 | 0.307 | 1.369 | 1.369(0.75-2.5) | 1.024 | 0.306 |
| Biliary disease | 1.372 | 0.235 | 3.942 | 3.942(2.487-6.249) | 5.83 | **<0.001** |
| ALT | -0.002 | 0.002 | 0.998 | 0.998(0.994-1.002) | -0.926 | 0.355 |
| FIB | -0.17 | 0.07 | 0.844 | 0.844(0.736-0.968) | -2.436 | **0.015** |
| ALB | -0.051 | 0.022 | 0.95 | 0.95(0.91-0.992) | -2.295 | **0.022** |
| PLT | -0.001 | 0.001 | 0.999 | 0.999(0.997-1.001) | -1.176 | 0.24 |
| PCT | 0.011 | 0.003 | 1.011 | 1.011(1.006-1.017) | 3.895 | **<0.001** |
| CRP | -0.002 | 0.001 | 0.998 | 0.998(0.996-1) | -1.551 | 0.121 |
| WBC | 0.013 | 0.011 | 1.013 | 1.013(0.992-1.035) | 1.153 | 0.249 |
| Abscess location | -0.026 | 0.151 | 0.975 | 0.975(0.725-1.31) | -0.17 | 0.865 |
| Multiple abscesses | 0.651 | 0.286 | 1.918 | 1.918(1.095-3.359) | 2.273 | **0.023** |
| Abscess diameter | 0.004 | 0.004 | 1.004 | 1.004(0.996-1.012) | 1.117 | 0.264 |
| Gas-containing abscess | -0.615 | 0.494 | 0.541 | 0.541(0.205-1.424) | -1.244 | 0.213 |
| Septic shock | 1.687 | 0.282 | 5.402 | 5.402(3.108-9.389) | 5.99 | **<0.001** |
| Pneumonia | -0.281 | 0.317 | 0.755 | 0.755(0.406-1.405) | -0.886 | 0.376 |
| Pleural effusion | 0.085 | 0.301 | 1.088 | 1.088(0.603-1.963) | 0.281 | 0.778 |
| Treatments | 0.283 | 0.224 | 1.327 | 1.327(0.855-2.058) | 1.262 | 0.207 |
| SOFA-2 score | 0.227 | 0.043 | 1.255 | 1.255(1.154-1.365) | 5.269 | **<0.001** |

T2DM:Type 2 Diabetes Mellitus;ALT:Alanine Aminotransferase;FIB:Fibrinogen;ALB:Albumin;PLT:Platelet;PCT:Procalcitonin;

CRP:C-reactive Protein;WBC:White Blood Cell;SOFA-2 score:Sequential Organ Failure Assessment 2.0 score.

**Supplementary Table 3. the predictors identified by Boruta algorithm and Lasso regression .**

| **Methods** | **Boruta** | **LASSO** |  |
| --- | --- | --- | --- |
| **Variables** | Age | Age |  |
|  | T2DM | T2DM |  |
|  | Malignant neoplasm | Malignant neoplasm |  |
|  | Cerebral infarction | Biliary disease |  |
|  | Biliary disease | FIB |  |
|  | ALT | PCT |  |
|  | FIB | CRP |  |
|  | ALB | Multiple abscesses |  |
|  | PLT | Gas-containing abscess |  |
|  | PCT | Septic shock |  |
|  | CRP | SOFA-2 score |  |
|  | WBC |  |  |
|  | Multiple abscesses |  |  |
|  | Abscess diameter |  |  |
|  | Gas-containing abscess |  |  |
|  | Septic shock |  |  |
|  | SOFA-2 score |  |  |

T2DM:Type 2 Diabetes Mellitus;ALT:Alanine Aminotransferase;FIB:Fibrinogen;ALB:Albumin;PLT:Platelet;PCT:Procalcitonin;

CRP:C-reactive Protein;WBC:White Blood Cell;SOFA-2 score:Sequential Organ Failure Assessment 2.0 score.

**Supplementary Table 4. Performance of XGBoost with different feature selection strategies.**

|  | **AUC** | **Accuracy** | **F1 score** | **Sensitivity** | **Specificity** | **Brier score** | **Precision (PPV)** | **NPV** |
| --- | --- | --- | --- | --- | --- | --- | --- | --- |
| **Final intersection set** | 0.891 | 0.894 | 0.736 | 0.667 | 0.958 | 0.091 | 0.821 | 0.910 |
| **LASSO** | 0.886 | 0.875 | 0.727 | 0.750 | 0.911 | 0.087 | 0.706 | 0.927 |
| **Univariate analyse** | 0.900 | 0.894 | 0.768 | 0.792 | 0.923 | 0.089 | 0.745 | 0.939 |
| **Boruta** | 0.890 | 0.898 | 0.771 | 0.771 | 0.935 | 0.085 | 0.771 | 0.935 |
| **Full feature set** | 0.886 | 0.894 | 0.753 | 0.729 | 0.941 | 0.086 | 0.778 | 0.924 |

AUC: area under the receiver operating characteristic curve;PPV:Positive Predictive Value;NPV:Negative Predictive Value.

**Supplementary Table 5. Five-fold cross-validation performance of the seven machine learning models (AUC).**

| **Model** | **Mean** | **Standard Deviation** |
| --- | --- | --- |
| **LM** | 0.8300 | 0.0168 |
| **DT** | 0.8305 | 0.0254 |
| **RF** | 0.8989 | 0.0171 |
| **XGBoost** | 0.8893 | 0.0144 |
| **Lightgbm** | 0.9251 | 0.0212 |
| **Catboost** | 0.9227 | 0.0169 |
| **MLP** | 0.8422 | 0.0216 |

LM: logistic regression model;DT: decision tree;RF: random forest;XGBoost: eXtreme gradient boosting; LightGBM: light gradient boosting machine;CatBoost:categorical boosting;MLP:multilayer perceptron;AUC: area under the receiver operating characteristic curve.


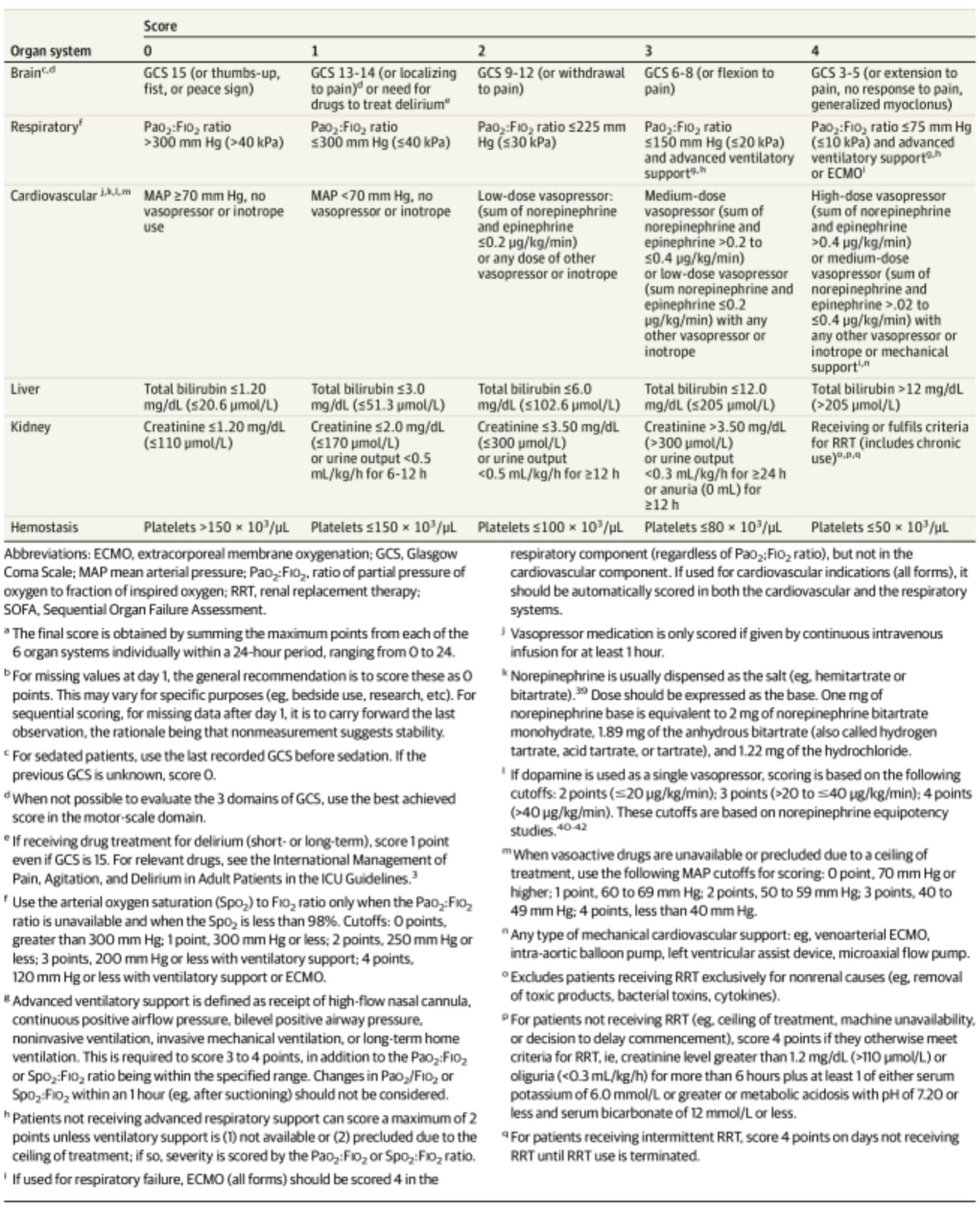


**Supplementary Figure 1. The SOFA-2 scoring criteria.**


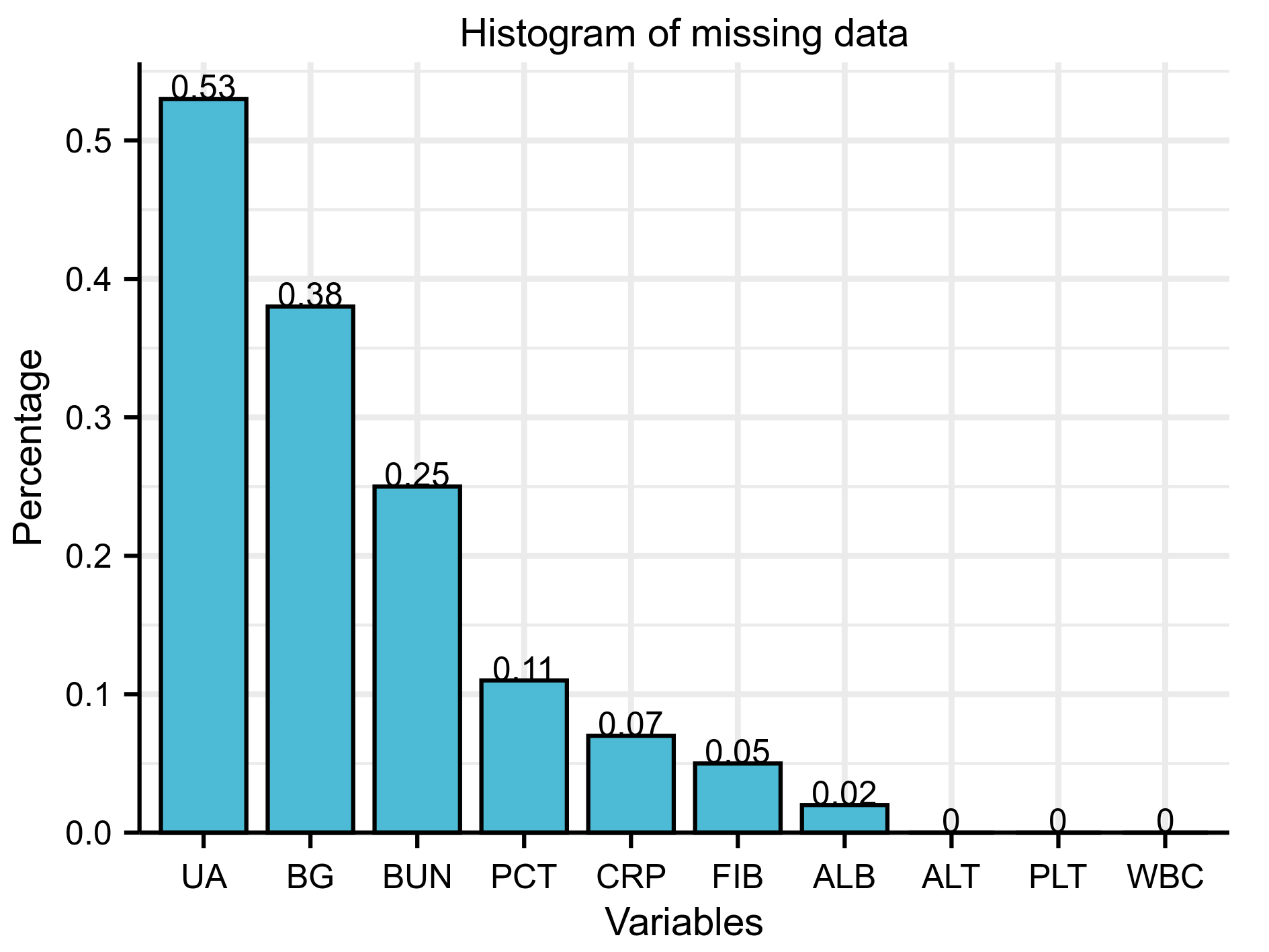


**Supplementary Figure 2. Histogram of missing data.**UA:Uric Acid;BG:Blood Glucose;BUN:Blood Urea Nitrogen;PCT:Procalcitonin;CRP:C-reactive Protein;FIB:Fibrinogen;ALB:Albumin;ALT:Alanine Aminotransferase;PLT:Platelet;WBC:White Blood Cell;


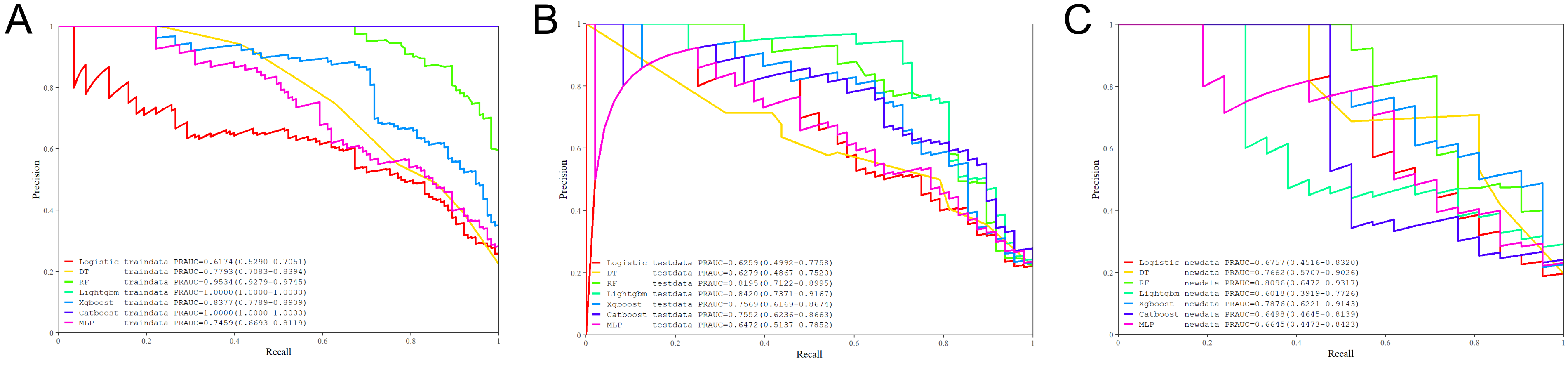


**Supplementary Figure 3. (A) Precision-Recall Curves for the Training set; (B) Precision-Recall Curves for the testing set;(C) Precision-Recall Curves for the external validation set.**LM: logistic regression model;DT: decision tree;RF: random forest;XGBoost: eXtreme gradient boosting; LightGBM: light gradient boosting machine;CatBoost:categorical boosting;MLP:multilayer perceptron


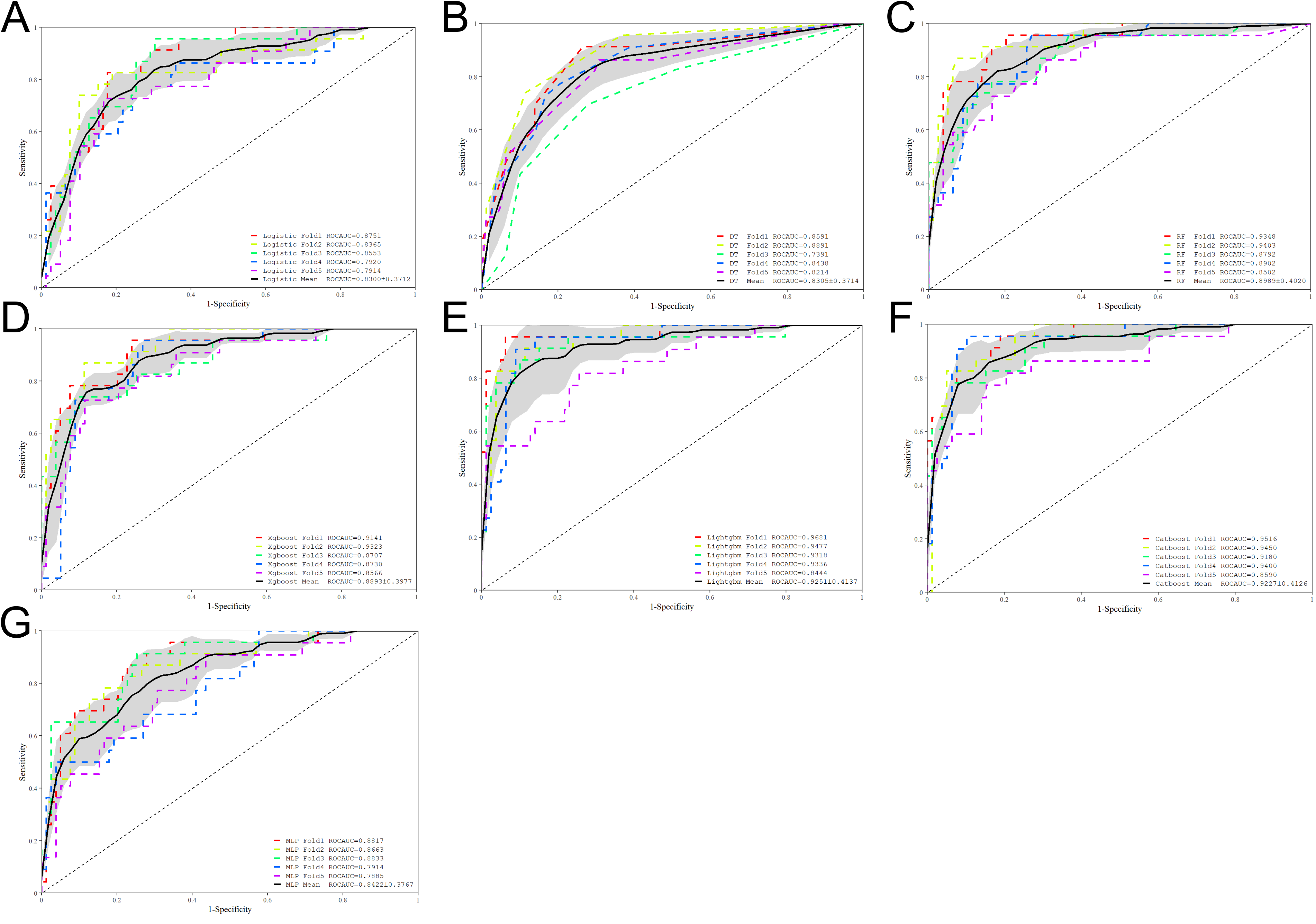


**Supplementary Figure 4. Five-fold cross-validation receiver operating characteristic (ROC) curves of the seven machine learning models on the training set.** Panels A-G correspond to the following models: (A) LM, (B) DT, (C) RF, (D) XGBoost, (E) LightGBM,(F)CatBoost,and (G) MLP. The colored dashed lines represent the ROC curves of each individual fold. The solid black line indicates the mean ROC curve across all five folds, and the gray shaded area represents the 95% confidence interval of the mean ROC curve. AUC values for each fold and the mean AUC are provided in the legend. LM: logistic regression model;DT: decision tree;RF: random forest;XGBoost: eXtreme gradient boosting; LightGBM: light gradient boosting machine;CatBoost:categorical boosting;MLP:multilayer perceptron.
